# Interferon-β Modulates the Innate Immune Response against Glioblastoma Initiating Cells

**DOI:** 10.1371/journal.pone.0139603

**Published:** 2015-10-06

**Authors:** Fabian Wolpert, Caroline Happold, Guido Reifenberger, Ana-Maria Florea, René Deenen, Patrick Roth, Marian Christoph Neidert, Katrin Lamszus, Manfred Westphal, Michael Weller, Günter Eisele

**Affiliations:** 1 Department of Neurology, University Hospital Zurich, Frauenklinikstrasse 26, CH-8091, Zurich, Switzerland; 2 Department of Neuropathology, Heinrich Heine University, Moorenstrasse 5, D-40225, Düsseldorf, Germany, and German Cancer Consortium (DKTK), German Cancer Research Center (DKFZ), Heidelberg, Germany; 3 Genomics and Transcriptomics Laboratory (GTL), Center for Biological and Medical Research (BMFZ), Heinrich Heine University, Moorenstrasse 5, D-40225, Düsseldorf, Germany; 4 Department of Neurosurgery, University Hospital Zurich, Frauenklinikstrasse 10, CH-8091, Zurich, Switzerland; 5 Department of Neurosurgery, University Hospital Hamburg-Eppendorf, Martinistraße 52, D-20246, Hamburg, Germany; University of Michigan School of Medicine, UNITED STATES

## Abstract

Immunotherapy targeting glioblastoma initiating cells (GIC) is considered a promising strategy. However, GIC are prone to evade immune response and there is a need for potent adjuvants. IFN-β might enhance the immune response and here we define its net effect on the innate immunogenicity of GIC. The transcriptomes of GIC treated with IFN-β and controls were assessed by microarray-based expression profiling for altered expression of immune regulatory genes. Several genes involved in adaptive and innate immune responses were regulated by IFN-β. We validated these results using reverse transcription (RT)-PCR and flow cytometry for corresponding protein levels. The up-regulation of the NK cell inhibitory molecules HLA-E and MHC class I was balanced by immune stimulating effects including the up-regulation of nectin-2. In 3 out of 5 GIC lines tested we found a net immune stimulating effect of IFN-β in cytotoxicity assays using NKL cells as effectors. IFN-β therefore warrants further investigation as an adjuvant for immunotherapy targeting GIC.

## Introduction

Despite multimodal approaches including surgery, radio- and chemotherapy, the efficacy of glioblastoma treatment remains limited, with an overall survival of about one year [1, 2]. New therapeutic options are needed to overcome this devastating disease and the use of immunotherapy is considered a promising option [3]. Possible strategies comprise immune check point inhibitors such as ipilimumab or nivolumab as well as active cellular immunotherapy or vaccination. The feasibility and safety of such approaches has been demonstrated in humans, however, no controlled trial has demonstrated a relevant and robust improvement of survival of glioblastoma patients (reviewed in [4–7]). Glioma cells express ligands for activating immune receptors like natural killer group 2 member D (NKG2D) or DNAX accessory molecule (DNAM)-1 [8–11], and should therefore be prone to recognition and elimination by the immune system. The insufficient response to immunotherapies may in part be due to immune evasive mechanisms in gliomas such as the down-regulation of ligands for NKG2D by transforming growth factor (TGF)-β [8, 10, 11] and certain miRNA species [12] or the expression of immune inhibitory proteins like HLA-E or—G [13–15].

New adjuvant strategies might help to overcome resistance to immune control in glioma. Interferons (IFN) have been explored as adjuvants for immunotherapies in cancer entities like melanoma or renal cell carcinoma in humans [16]. The type I IFN (IFN-α and IFN-β) and type II IFN, such as IFN-γ, are the best characterized and clinically most relevant IFN. Stimulation with IFN leads to an up-regulation of *major histocompatibility complex (MHC) -I and -II* gene expression as well as antigen presentation in dendritic cells (DC) [17–19], and glioma cells [11, 13, 15, 18], enhancing antigen-presenting capabilities. Moreover, recent reports in mice and humans describe an immune-independent direct anti-tumor activity of IFN-β [20]. Glioma cells might be sensitized to the alkylating agent temozolomide (TMZ) [21, 22], and combined therapy of IFN-β and TMZ resulted in a favorable outcome in patients with tumors with O^6^-methylguanine DNA methyltransferase (MGMT) promotor methylation [23]. Modulation of tumor vasculature [24], down-regulation of MGMT expression [21, 23] and induction of apoptosis by IFN-β independently of MGMT-mediated resistance to temozolomide [25–27] have been discussed as mechanisms of this anti-glioma effect. Based on these multi-directional activities, IFN-β warrants further evaluation as an adjuvant for anti-glioma immunotherapies, possibly bridging innate and adaptive immune responses [28].

A crucial issue for an effective immunotherapy of glioma is the definition of the target. Glioma cells with stem cell-like properties are supposed to be essential for tumor initiation and relapse. These glioma-initiating cells (GIC) are defined by their stem cell-like properties of self-renewal, multipotency and tumorigenicity in immunodeficient mice, forming tumors resembling the initial human tumors [29, 30]. We recently identified the atypical human leukocyte antigen (HLA-)-E as an immune-compromising factor in GIC [13]. The interaction of HLA-E with its receptor, the dimer CD94/NKG2A, leads to inhibition of the lytic activity of natural killer (NK) cells towards GIC. Moreover, a disintegrin and metalloproteinase (ADAM) 10 and 17 cleave the UL16 binding protein (ULBP) 2 from the cell surface of GIC. This hampers NK cell activity against GIC since ULBP2 is a ligand of NKG2D. The other NKG2D ligands that may be expressed on GIC are MHC class I chain-related antigen (MIC)A and -B and UL16 binding protein (ULPB)1-6 [8, 11]. Furthermore, nectin-2 and poliovirus receptor (PVR), ligands of DNAM–1, are supposed to be important immune-stimulating proteins present on GIC [11].

Here we define the net effect of IFN-β treatment on the innate immunogenicity of GIC.

## Materials and Methods

### 2.1 Materials and cell lines

The GIC lines GS-2, GS-5, GS-7, GS-8 and GS-9 have been previously characterized for stem cell properties [31]. LNT–229 glioma were kindly provided by N. de Tribolet (Lausanne, Switzerland) [32] and cultured as described [27]. All GSC lines were cultured in 75 cm^2^ culture flasks and maintained in neurobasal medium with B–27 supplement (20 μl/ml) and glutamax (10 μl/ml) from (all Invitrogen) fibroblast growth factor (FGF)-2, epidermal growth factor (EGF) (20 ng/ml each; Peprotech, Rocky Hill, PA) and heparin (32 IE/ml; Ratiopharm, Ulm, Germany). Stem cell factors were supplemented twice a week, complete medium changed once a week. Cells were passaged when spheres reached an estimated diameter of 500 μm or an estimated density of 5 x 10^4^ cells/cm^2^. Spheres were dissociated mechanically and enzymatically. Briefly, we spun down the cells and resuspended the pellet in 1 ml accutase (PAA, Wien, Austria). After mechanical dissection by pipetting up and down, we incubated the cells at 37°C for 5 minutes. From previous work we know that accutase does not alter the expression level of NKG2DL on the cell surface of glioma cells [10, 33]. The NK cell line NKL was a gift from M. Robertson (Indianapolis, IN) [34] and cells were cultured in RPMI 1640 medium (PAA) containing 15% fetal calf serum (FCS), 2 mM L-glutamine (Gibco Life Technologies, Paisley, UK), penicillin (100 IU/ml)/streptomycin (100 mg/ml) (Gibco), 1 mM sodium pyruvate and 100 U/ml interleukin 2 (Peprotech).

IFN-β1b was purchased from AbD Serotec (Dusseldorf, Germany) and reconstituted to a concentration of 10^6^ IU/ml with distilled H_2_O. Cell surface expression of immuneregulatory proteins was assessed with the following monoclonal antibodies (mAbs): HLA-E (clone 3D12; unconjugated (dilution 1:200) and APC-conjugated (dilution 1:50) mouse IgG1; eBioscience, Vienna, Austria [13]), MHC-I (clone W6/32; mouse IgG2a, Biologend, Uithoorn, Netherlands, dilution 1:1000 [13]), PVR and nectin-2 (clones SKII.4, dilution 1:50 and TX31; mouse IgG1, dilution 1:100 both Biolegend. Optimal conditions were determined by dilution row experiments). Antibodies for MICA, MICB and ULBP1-3 were provided by A. Steinle (Frankfurt, Germany) [8] and diluted at 1:100 [35]. For NK cell staining, the following antibodies were used, and optimal conditions were determined by dilution row experiments: CD94 (Clone REA113, APC-conjugated mouse IgG1, Miltenyi Biotech, Bergisch-Gladbach, Germany, dilution 1:500), NKG2A (clone REA110, PE-conjugated mouse IgG1, Miltenyi Biotech, dilution 1:200), NKG2C (clone 134591, Alexa Fluor^®^ 488 conjugated mouse IgG1, R&D Systems, Minneanapolis, MA, dilution 1:100), NKG2D (clone 1D11, PE-conjugated mouse IgG1, Biolegend, dilution 1:1000) and DNAM–1 (ab89394, unconjugated mouse IgG1, Abcam, Cambridge, UK, dilution 1:1000). Unconjugated IgG1 (clone MOPC 21; Sigma-Aldrich, Buchs, Switzerland) and IgG2a (clone MOPC–173; Biolegend) or conjugated isotype-matched mAbs were used as controls (clone MOPC 21; PE-conjugated mouse IgG1, Sigma-Aldrich; Clone: P3.6.2.8.1, APC-conjugated IgG1, eBioscience; clone MOPC 21; Alexa Fluor^®^ 488 conjugated IgG1, Biolegend). The PE-conjugated goat anti-mouse IgG (Dako, Freiburg, Germany) was used as secondary antibody where appropriate in a dilution of 1:20.

### 2.2 Flow cytometry

GIC growing as spheres were mechanically dissociated by pipetting and dissolved in Accutase (PAA, Vienna, Austria) to get a single cell suspension. Adherent cells were detached using Accutase. The cells were preincubated in PBS with 2% fetal calf serum (FCS) and stained with specific mAb or matched mouse Ig isotype for 30 min on ice. For unconjugated mAB, staining was followed by incubation with a PE-conjugated secondary antibody for 30 min respectively. The cells were washed with PBS containing 2% FCS. Flow cytometry was performed with a CyAn ADP Analyzer (Beckman Coulter, Nyon, Switzerland). Specific fluorescence index (SFI) was calculated by dividing median fluorescence obtained with the specific antibody by median fluorescence obtained with the control antibody.

### 2.3 Real-Time PCR

RNA was prepared from the cell lines using NucleoSpin RNA II Kit (Macherey-Nagel, Düren, Germany). Subsequently, cDNA was generated using SuperScript II Reverse Transcriptase (Invitrogen AG, Basel, Switzerland). RNA and DNA concentrations were determined with the NanoDrop spectrometer (Thermo Scientific, Waltham, MA). cDNA amplification was monitored using SYBRGreen chemistry on the ABI PRISM7000 Sequence Detection System (Applied Bio-Systems, Weiterstadt, Germany) with the following specific primers (forward/reverse): GAPDH: 5’-CTCTCTGCTCCTCCTGTTCGAC–3’ (450–469), 5’-TGAGCGATGTGGCTCGGCT–3’ (636–619); HLA-E: 5’-GGGACACCGCACAGATTTT–3’ (266–284), 5’CTCA-GAGGCATCATTTGACTTTT (519–497)[14]. Data analysis was done using the ddCT method for relative quantification. Briefly, threshold cycles (CT) for GAPDH RNA (reference) and HLA-E (sample) were determined in duplicate. The expression levels were determined following the formula: rI = 2^-[C_T_HLA-E—C_T_ GAPDH]^ and standardized to normal brain cDNA (Amsbio, Abingdon, UK).

### 2.4 RNA interference

To silence gene expression, glioma cells were transiently transfected with siRNA molecules targeting specifically HLA-E [14]: (535–553): 5’-GCCUACCUGGAAGACACAU(dTdT)-3’ and 5’-AUGUGUCUUCCA-GGUAGGC(dTdT)-3’, for nectin-2 gene silencing ON-TARGETplus nectin-2 siRNA (Dharmacon, Lafayette, CO) was applied. ON-TARGETplus Non-Targeting Pool siRNA (Dharmacon) was used as a control in all gene silencing experiments. Glioma cells were transfected with 2 nmol siRNA through electroporation with one pulse (1500 mV, 10 ms), using a Neon^®^ Transfection system (Invitrogen). The result of gene silencing was verified by flow cytometry at 24 h after transfection.

### 2.5 Immune cell cytotoxicity assay

We used a flow cytometry-based cytotoxicity assay which employs the membrane dye PKH–26 and the viability dye ToPro3 [36]. The target cells were stained with PKH–26 membrane dye. This enables the discrimination of the target cells from the effector cells in the subsequent lysis assay. Briefly, the target cells were incubated with PKH–26 dissolved in Diluent C (1:250; all from Sigma-Aldrich) for 3 min. The staining was stopped by washing with RPMI 1640 medium containing 10% FCS. Then the effector and target cells were co-incubated at varying effector to target (E:T) ratios for 3 h. Immediately prior to flow cytometry, the viability dye ToPro3 (Invitrogen) was added. The percentage of target cell lysis was determined by flow cytometry using the CyAn ADP Analyzer (Beckman Coulter). Specific lysis was assessed by subtraction of background lysis.

### 2.6 Microarray-based gene expression profiling

The technical details have been described elsewhere [27]. Array records are deposited under GEO accession number GSE53213.

Additional functional GeneOntology (GO) classification of differentially expressed genes (fold change >2x) was performed using GeneSpring GX (version 12.5; Agilent Technologies). The corrected enrichment p-value cutoff was set to 0.1. Hierarchical clustering of differentially expressed transcripts within selected GO terms was done employing Euclidian similarity measures and Ward´s linkage.

### 2.7 STRING inquiry

For analysis of functional gene interactions and visualization, data were entered into the *Search Tool for the Retrieval of Interacting Genes/Proteins* (STRING) Version 9.1 at http://string-db.org [37].

### 2.8 Statistical analysis

The experiments were performed 2 to 3 times as indicated. Statistical significance was calculated by student`s two-tailed t-test (* p<0.05; ** p<0.01). Adobe illustrator^®^ CS 6 (version 16.0, Adobe Systems) was used for design of graphics.

## Results

### 3.1 Expression profiling reveals regulation of immune regulatory genes in GIC upon stimulation with IFN-β

**Fig 1:**
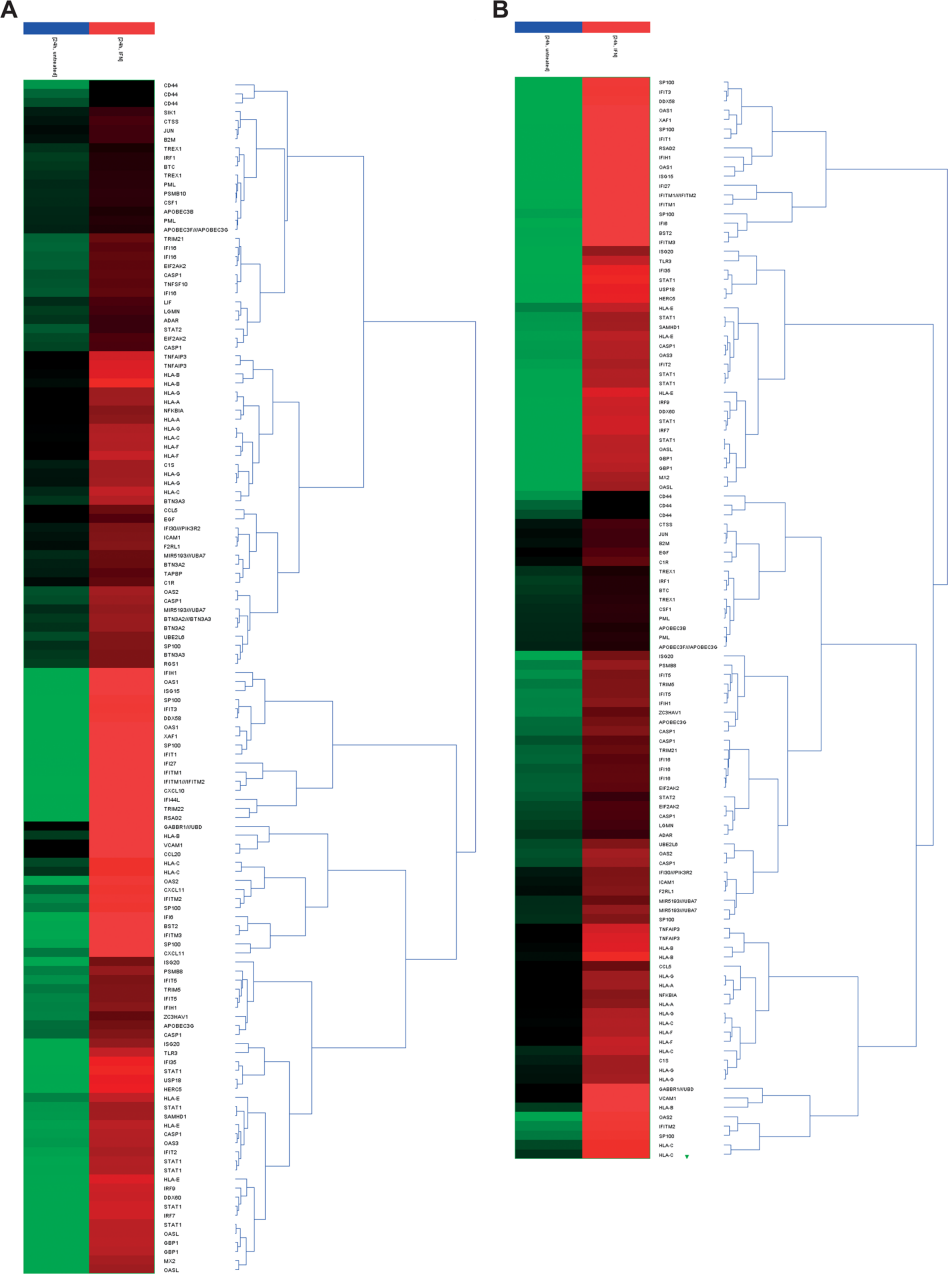
Hierarchical clustering of differentially expressed transcripts. GS-9 cells were treated with IFN-β (300 U/ml, 24h) and the changes in the transcriptome were assessed using Affymetrix chip-based expression profiling. Immune regulatory genes were identified by functional GeneOntology analysis of differentially expressed transcripts (fold change (FC)>2x GO:0006955 Immune response (A) and GO:0045087 regulation of innate immune response (B)). Data is shown as heat map, the color code represents the normalized log expression values.

Based on our previous microarray gene expression profiling experiments [27] we analyzed the changes in the RNA expression pattern of immune regulatory genes upon treatment with IFN-β in LNT–229 cells and the GIC lines GS-2 and GS-9. Genes involved in the immune response in general were selected according to the *Biological Process Ontology Guidelines* (GO:0006955, http://www.geneontology.org) [38] (Fig 1 and S3 Fig). Moreover, we assessed for genes involved in the regulation of the innate immune response (GO:0045088) (Fig 1 and S3 Fig). Furthermore, data were analyzed and visualized employing *Search Tool for the Retrieval of Interacting Genes/ Proteins* STRING (S1A–S1C Fig).

The transcripts encoding typical (HLA-A, -B, -C) and atypical MHC proteins (HLA-E, -F and -G), as well as proteins of the immuno-proteasome (*TAP1*, *PSMB8* and *PSMB9)* were increased upon stimulation with IFN-β. Moreover, genes encoding chemokines, toll-like receptors or death receptor ligands were up-regulated (Fig 1, S3 Fig and summarized in S1 Table).

### 3.2 Alteration of NK cell inhibiting ligand-receptor systems in GIC upon treatment with IFN-β

**Fig 2:**
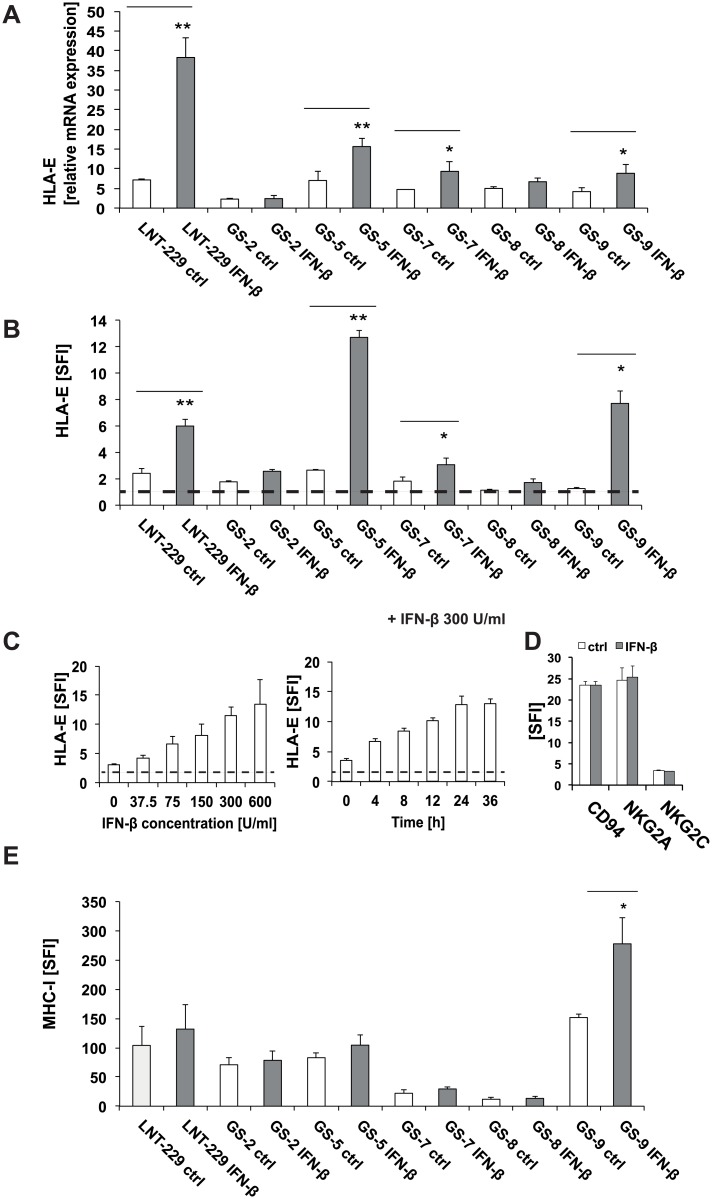
IFN-β modulates the expression of NK cell inhibiting ligand-receptor systems in GIC. A, B. *HLA-E* mRNA and cell surface protein levels in LNT–229 and GIC were analyzed following treatment with IFN-β (300 U/ml, 24 h) by RT-PCR and flow cytometry. C. GS-5 cells were treated with increasing concentrations of IFN-β or for various time periods (300 U/ml) as indicated. HLA-E surface levels were assessed by flow cytometry. (D). The cell surface levels of NKG2A and NKG2C and their dimerization partner CD94 (D) and of MHC class I (E) were assessed by flow cytometry. RT-PCR data are expressed relative to normal brain cDNA and represent mean relative expression ± SD from 3 independent experiments. Flow cytometry data represent mean SFI values ± SD from 2 to 3 independent experiments (SFI = 1 is marked with a dotted line in B, C, D. (** p<0.01, * p<0.05; student`s two tailed t-test).

We have previously defined a prominent NK cell suppressive effect of HLA-E expression by GIC [13]. Here we confirmed the *HLA-E* gene expression in LNT–229 as well as GS-2, GS-5, GS7, GS8 and GS-9. *HLA-E* mRNA was up-regulated significantly in LNT–229, GS-5, GS-7 and GS-9 cells (Fig 2A) following stimulation with IFN-β. Accordingly, the cell surface levels of HLA-E were significantly increased in LNT–229, GS-5, GS-7 and GS-9 cells following stimulation with IFN-β as assessed by flow cytometry (Fig 2B). In GS-2 and GS-8, HLA-E regulation at the mRNA and protein levels was statistically not significant (Fig 2A and 2B). The regulation of HLA-E by IFN-β was concentration- and time-dependent with peak HLA-E levels after 24 h using a concentration of 300 U/ml IFN-β in GS-5 (Fig 2C). In contrast, the expression of the HLA-E receptors NKG2A and NKG2C as well as their common dimerization partner CD94 on the NK cell line NKL remained unaltered in response to IFN-β at the same conditions (Fig 2D). The NK cell inhibiting classical HLA-molecule MHC-I was up-regulated only in GS-9, but remained unaltered in LNT–229, GS-2, GS-5, GS-7 and GS-8 cells upon treatment with IFN-β (Fig 2E).

### 3.3 Alteration of the expression of activating ligand-receptor systems in GIC and NK cells upon treatment with IFN-β

**Fig 3:**
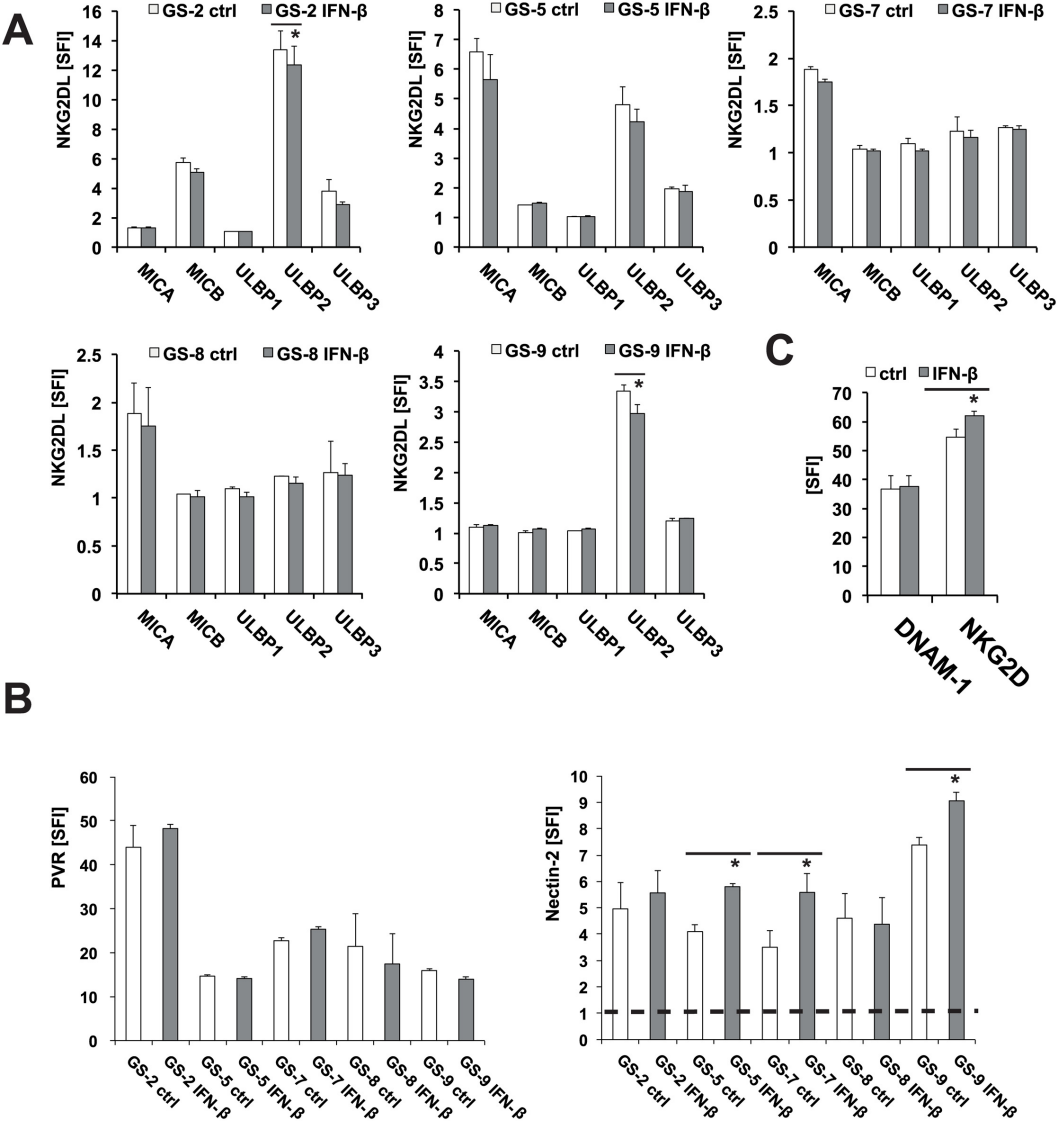
IFN-β modulates the expression of NK cell activating ligand-receptor systems in GIC. The levels of MICA, MICB, ULBP1, ULBP2 and ULBP3 (A) and of nectin-2 and PVR (B) were assessed by flow cytometry following treatment with IFN-β (300 U/ml, 24 h). C. The levels of NKG2D and DNAM–1 on NKL cells were assessed following treatment with IFN-β (300 U/ml, 24 h) by flow cytometry (note an almost complete overlay of the flow cytometry curves of treated and untreated cells). Flow cytometry data represent mean SFI values ± SD from 3 independent experiments (SFI = 1 is marked with a dotted line) (** p<0.01, * p<0.05; student`s two tailed t-test).

NK cell function is regulated via a balance of inhibiting and activating signals. Thus, we next assessed the effect of IFN-β on the expression of activating NK cell ligands MICA, MICB, ULBP1-3 and of nectin-2 and PVR on the cell surface of GIC which might counteract the immune inhibiting effect of HLA-E up-regulation. The levels of the corresponding receptors NKG2D and DNAM–1 were assessed on NKL cells. We found a down-regulation of ULBP2 in GS-2 and GS-9 cells upon treatment with IFN-β, whereas MICA, MICB, ULBP1 and ULBP3 remained unaltered (Fig 3A). The likewise immune stimulating DNAM–1 ligands nectin-2 and PVR were detected on the cell surface of GS-2, GS-5, GS-7, GS-8 and GS-9 by flow cytometry. Treatment with IFN-β led to an up-regulation of nectin-2 in GS-5, GS-7 and GS-9, but not in GS-2 and GS-8 while PVR remained unaltered in all cell lines tested (Fig 3B). While the expression of the activating immune cell receptor NKG2D was significantly up-regulated, DNAM–1 levels on NKL cells did not show any changes following stimulation with IFN-β (Fig 3C). The modulation of the expression of selected immune regulatory genes and proteins following IFN-β treatment is summarized in S2 Table.

### 3.4 The immunogenicity of GIC is modulated upon stimulation with IFN-β

**Fig 4:**
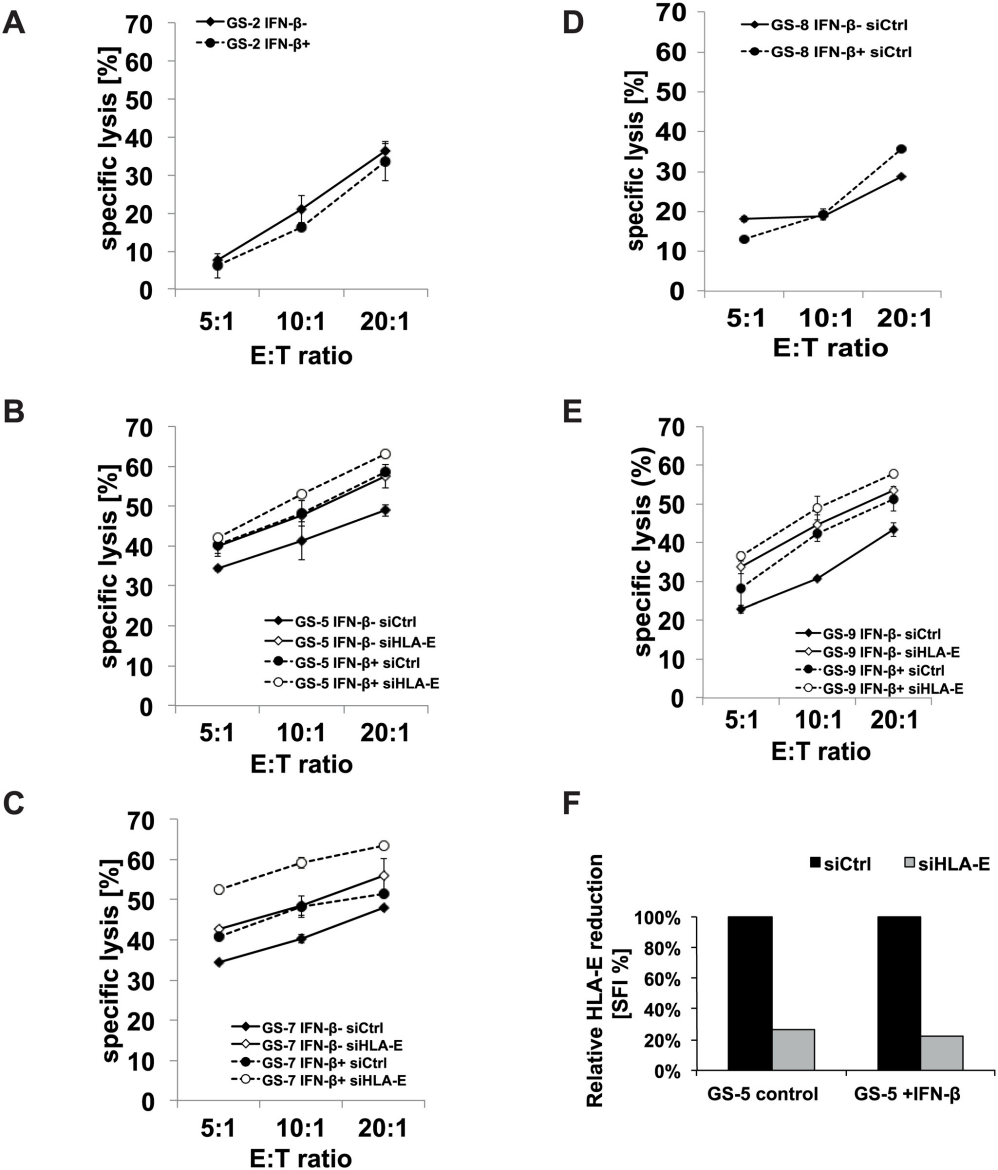
NKL cell-mediated cytotoxicity assays determine the net effect of IFN-β treatment on GIC. A-E. GS-2, GS-5, GS-7, GS-8 or GS-9 cells were used as targets in cytotoxicity assays and treated with IFN-β (300 U/ml; 24 h) prior to the cytotoxicity assay with NKL cells as effectors (1 out of 2 independent experiments shown). GS-5 and GS-9 cells were additionally transfected with siRNA targeting *HLA-E* or non-targeting siRNA. Specific lysis was calculated by subtraction of background lysis. In F, HLA-E cell surface levels after HLA-E gene silencing were assessed by flow cytometry (one representative experiment shown).

Having assessed the regulation of anti- and pro-immunogenic proteins, we next assessed the net effects of IFN-β treatment on the innate immunogenicity of GS-2, GS-5, GS-7, GS-8 and GS-9 cells. While the susceptibility towards NK cell-mediated lysis of GS-2 and GS-8 cells remained largely unaltered, the cytotoxicity towards GS-5, GS-7 and GS-9 cells was enhanced upon stimulation with IFN-β as assessed by a flow cytometry-based cytotoxicity assay (Fig 4A–4E). Moreover, we investigated the role of enhanced HLA-E cell surface levels on NK cell-mediated immune recognition in these two GIC lines exhibiting altered immunogenicity upon stimulation with IFN-β. *HLA-E* expression was silenced using RNA interference following incubation with IFN-β. The silencing of *HLA-E* gene expression led to a reduction of more than 70% of HLA-E cell surface levels, as ascertained by flow cytometry (Fig 4F). As expected, NKL-mediated lysis was enhanced in GS-5, GS-7 and GS-9 following silencing of *HLA-E* expression. The increased lysis following treatment with IFN-β was further enhanced by HLA-E-gene silencing indicating that the IFN-β-induced up-regulation of HLA-E counteracts a net immune enhancing effect of IFN-β (Fig 4B, 4C and 4E). Pretreatment of NKL cells with IFN-β did not influence their cytotoxic activity towards GS cells (S2A and S2B Fig). Therefore, effects of IFN-β on NK cell receptors seem to be negligible in this context.

**Fig 5:**
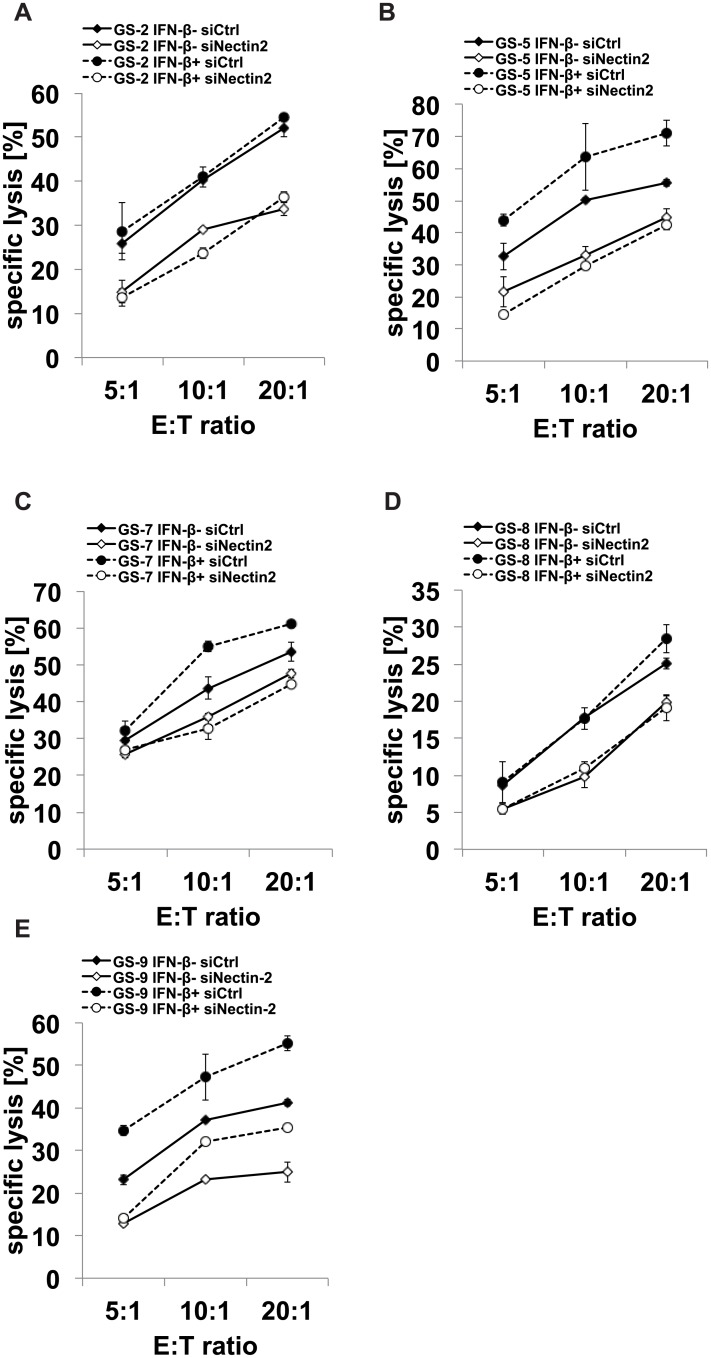
Gene silencing of *nectin-2* decreases GIC susceptibility to NKL cell-mediated cell death. GS-2, GS-5, GS-7, GS-8 or GS-9 cells were transfected with siRNA targeting *nectin-2* or non-targeting siRNA as a control and treated with IFN-β (300 U/ml, 24 h) prior to a cytotoxicity assay using NKL as effectors. In A-E, the results from the cytotoxicity assay are shown (1 out of 2 to 3 independent experiments shown). The cytotoxicity assays were run at several effector to target (E:T) ratios in duplicates. In F-J, the respective nectin-2 cell surface levels following n*ectin-2* gene silencing were assessed by flow cytometry. Specific lysis was calculated by subtraction of background lysis.

We next assessed the role of the up-regulation of nectin-2 for the net immune regulating effect of IFN-β in GS-9 cells. *nectin-2* expression was silenced using sequence-specific RNA interference in GS-2, GS-5, GS-7, GS-8 and GS-9 cells. The effect of gene silencing on nectin-2 cell surface expression was assessed by flow cytometry, followed by functional lysis assays with NKL effector cells. nectin-2 levels were reduced by 70–90% in IFN-β pretreated cells. In GS-5 and GS-7 cells, the immune activating effect of IFN-β was abolished by *nectin-2* gene silencing, indicating that enhanced immunogenicity is primarily mediated by nectin-2 in these cells (Fig 5B and 5C). In GS-9 cells, immune activation was only partially reverted, indicating involvement of additional stimulating ligands (Fig 5E). In GS-2 and GS-8, the silencing of *nectin-2* led to reduced cell lysis, however, regardless of treatment with IFN-β.

## Discussion

Immunotherapy is a promising approach to improve the outcome of glioblastoma patients. However, the choice of the target and the use of appropriate adjuvants are crucial. Potential future adjuvants like imiquimod or polyinosinic:polycytidylic acid lead, among other mechanisms, to enhanced expression of IFN-β. Recent uncontrolled clinical trials indicate a survival benefit for glioblastoma patients when adding IFN-β to radio- and chemotherapy [23, 39]. Type I IFN up-regulate the expression of co-stimulatory molecules like B7-1/B7-2 and may therefore enforce the adaptive immune response [40, 41]. However, they might also boost innate immune responses. In hepatitis C patients, treatment with IFN-α enhanced killing of infected cells in a DNAM–1 and NKG2D dependent manner [42]. Notably, IFN-β has been used as an adjuvant in several mouse immunotherapy models. An adenoviral vector encoding for IFN-β led to a CD8+ T cell dependent reduction of mesothelioma growth [43]. Furthermore, IFN-β enhanced tumor immunity in a vaccine model for leukemia through induction of WT1-specific cytotoxic T cells and enhancement of NK cell activity [44]. In glioma models, IFN-β promoted anti-glioma cytotoxic T cell as well as NK cell activity [45] and glioma cells transfected with IFN-β gene showed enhanced immune recognition via DC and cytotoxic T cells in vitro and in vivo [46]. These data strengthen the potential usefulness of IFN-β as an adjuvant for immunotherapy of tumors. The induction of typical and atypical MHC-proteins is a conserved hallmark of type I and type II interferons [19].

In the present work, we evaluated the changes of the innate immunologic phenotype of glioblastoma stem-like cells mediated by IFN-β and define implications for its clinical use as an adjuvant for immunotherapy targeting GIC. Employing microarray-based expression profiling, we identified alterations in the expression of several overall immune response and innate immune response genes, in a panel of well-defined GIC upon treatment with IFN-β (Fig 1 and S1 Fig). These data revealed an up-regulation of multiple immune regulatory genes including the atypical MHC proteins HLA-E, -F and -G, genes of the antigen presenting machinery (e.g. TAP1, PSMB8 and PSMB9), chemokines (e.g. CCL20) and toll like receptors (e.g. TLR3) (Fig 1, S1 Fig, S1 Table). The up-regulation of HLA-E as a potentially immunosuppressive factor is contrasted by enhanced surface levels of the immune activating molecule nectin-2 while the level of NKG2D ligands remained largely unaltered (Figs 2 and 3).

Since NK cell function is regulated via a balance of inhibiting and activating signals, we performed NK cell lysis assays to assess the net functional effect of IFN-β on the innate immunogenicity of GIC. Despite the up-regulation of HLA-E, we could not find a net inhibitory effect of IFN-β on NK cell-mediated cytotoxicity towards GIC. NKL cell-mediated cytotoxicity towards 3 of 5 GIC lines was even increased upon IFN-β treatment, while it remained unchanged in two cell lines (GS-2 and GS-8) (Fig 4A). A net immune stimulatory effect of IFN-β was further enhanced upon gene silencing of HLA-E using RNA interference (Fig 4B and 4C). This indicates that other immune-modulating mechanisms conferred by IFN-β might override the HLA-E mediated immune inhibition. We already observed a heterogeneous modulation of innate immunity towards GIC by IFN-γ. Despite a significant up-regulation of HLA-E and MHC-I, innate immunogenicity was reduced in only one (GS-5) of the two cell lines (GS-5 and GS-7) investigated [13]. However, the comparability between type I and II IFN is limited and should be considered with caution. In the GIC cell lines investigated here, the concurrent up-regulation of immune stimulatory proteins like nectin-2 counteracted the detrimental effect of an up-regulation of HLA-E. In GS-5 and GS-7, we provide direct evidence for the importance of nectin-2 for the immune stimulating effect of IFN-β. Both cell lines show enhanced immunogenicity following treatment with IFN-β and this effect was reversed upon nectin-2 gene silencing (Fig 5B and 5C). Treatment of GS-9 cells with IFN-β led to a similar up-regulation of nectin-2 on the cell surface compared to GS-5 and GS-7. In contrast, *nectin-2* gene silencing did not reverse the immune stimulating effects from IFN-β in GS-9 to the same degree like in GS-5 and GS-7. The in general sufficient however indeed incomplete silencing of nectin-2 expression levels might play a role, but the involvement of other receptor-ligand systems in this particular GS line might better explain these differences. Regarding the NKG2D system, we did not find alterations of the expression levels of either the ligands or the receptor, NKG2D, upon treatment with IFN-β in these GS lines (Fig 3A and 3C). This is also true for the other well-known DNAM–1 ligand PVR and the receptor itself. The natural cytotoxicity receptors NKp30, NKp44 and NKp46 could play a role in GS-9 cells, but the respective ligands are still insufficiently characterized.

Compared to GS-5, GS-7 and GS-9, the GIC lines GS-2 and GS-8 showed only marginal changes in the expression levels of the immune regulatory proteins investigated here upon treatment with IFN-β, except for a significant down-regulation of ULBP–2 levels in GS-2 (Figs 2 and 3). In GS-2 and GS-8, IFN-β did not alter the expression levels of nectin-2. Accordingly, these 2 cell lines show no difference in NK cell-mediated lysis upon treatment with IFN-β. Nevertheless, nectin-2 is important for the immunogenicity of these cells in general since the silencing of *nectin-2* lead to reduced cell lysis, again regardless of treatment with IFN-β. These results strengthen the importance of nectin-2 for the immune stimulating effect of IFN-β observed in GS-5 and GS-7. The failure of IFN-β to change the immunogenicity of GS-2 cells towards NKL cells cannot be explained with a general unresponsiveness of these cell lines taking into account major changes in the expression of several immune regulatory genes upon treatment with IFN-β and expression of IFN-β receptors (S1 Table and Happold et al. [27]).

Gene expression analysis revealed 2 different subtypes of the GIC lines used here [31]. GS-5, GS-8 and GS-9 were characterized by the expression of neurodevelopmental genes and a full stem-like phenotype while GS-2 and GS-7 exhibited an expression signature enriched for extracellular matrix-related genes with a restricted stem-like phenotype. GIC subtypes might therefore also differ in immunological phenotypes.

Altogether, IFN-ß shifts the balance between immune-inhibitory and immune-stimulatory cell surface markers towards increasing immunogenicity in most experimental systems but in a heterogeneous fashion.

Our data indicate that IFN-β has at least no detrimental effect on the innate immunogenicity of GIC and thus warrants further investigation as an adjuvant for immunotherapy targeting GIC. Since the overall functions of IFN-β are more complex, future investigations will focus on IFN-β-mediated modulation of adaptive immune responses towards GIC.

## Supporting Information

S1 Fig STRING analysis of the modulation of immune regulatory genes upon treatment with IFN-β. A-C. LNT–229, GS-2 or GS-9 cells were treated with IFN-β (300 U/ml, 24 h) and the changes in the transcriptome were assessed using Affymetrix Gene Chip HG-U133A 2.0 arrays (Affymetrix, Santa Clara, CA) as reported (Happold et al. 2014). Immunoproteins were identified according to *Biological Process Ontology Guidelines* and entered into the STRING tool. For full names see S2 Table.
(EPS)

S2 Fig Pretreatment of NKL cells with IFN-β does not alter their immunoreactivity. A, B. NKL, GS-2 (A) or GS-9 (B) cells were treated with IFN-β (300 U/ml, 24 h) prior to use as effector or target cells, respectively, in a cytotoxicity assay (n = 2). The cytotoxicity assays were run at several effector to target (E:T) ratios in duplicates. Specific lysis was calculated by subtraction of background lysis.
(EPS)

S3 Fig Expression profiling of immune response genes. GS-2 (A, B) and LNT–229 (C, D) cells were treated with IFN-β (300 U/ml, 24 h) and the changes in the transcriptome were assessed using Affymetrix chip-based expression profiling. Results are presented as heat map.
(PDF)

S1 Table Summary of the modulation of the expression of immune regulatory genes from RNA microarray. LNT–229, GS-2 or GS-9 cells were treated with IFN-β (300 U/ml, 24h) and the changes in the transcriptome were assessed using Affymetrix chip-based expression profiling. Immune regulatory genes were identified according to *Biological Process Ontology Guidelines*. Results are summarized in a table, showing the official gene symbol, full name, Probe set ID (= Gene accession number) and the grade of up-regulation compared to untreated controls.
(PDF)

S2 Table Summary of the modulation of the expression of selected immune regulatory proteins in GIC and NKL cells by IFN-β. The modulation of immune regulatory genes or surface proteins upon treatment of GSC and NKL with IFN-β is visualized by arrows, that indicate up- (**↑**), down- (**↓**) or unregulated (**→**) expression. Only marginal, but formally significant alterations of expression or surface levels were marked with brackets.
(EPS)
