# Supplementary material for: Interferon-β Modulates the Innate Immune Response against Glioblastoma Initiating Cells
Source: PLoS One. 2015 Oct 6;10(10):e0139603. doi: 10.1371/journal.pone.0139603 (PMC4595134; doi:10.1371/journal.pone.0139603)

Supp. Figure 3 A

GS-2
Immune response
GO:0006955

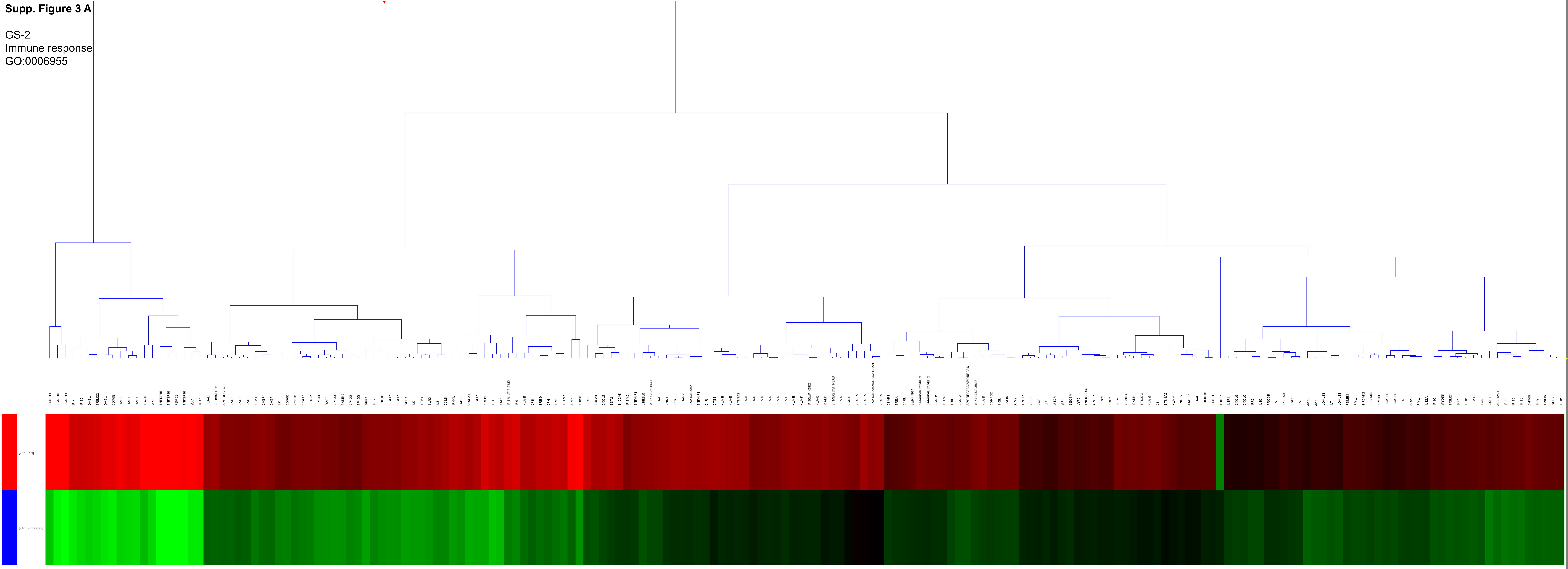

Supp. Figure 3 B

GS-2 innate immune response
GO:0045087

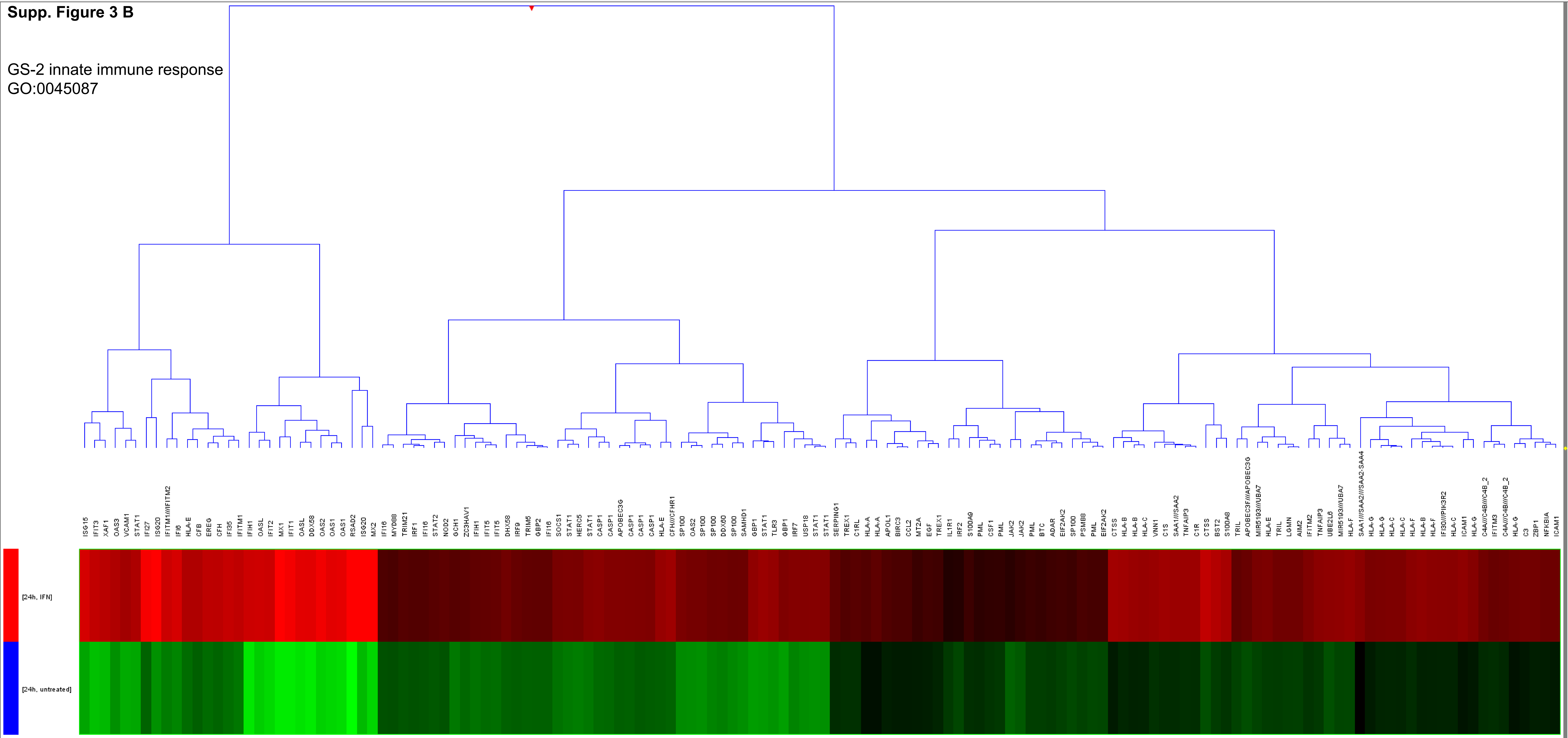

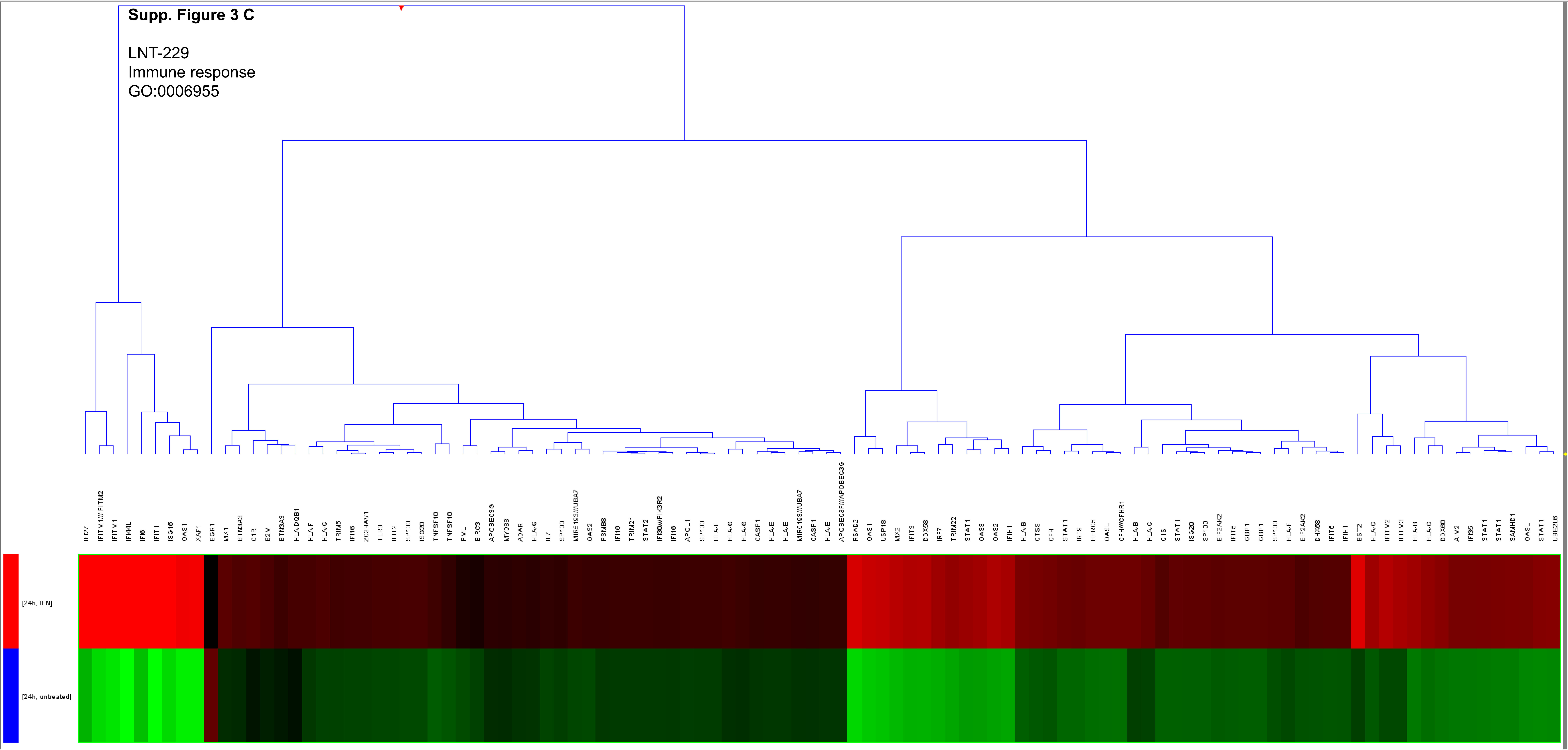
Supp. Figure 3 C
LNT-229
Immune response
GO:0006955
IFI27
IFITM1///IFITM2
IFITM1
IFI44L
IFI6
IFIT1
ISG15
OAS1
XAF1
EGR1
MX1
BTN3A3
C1R
B2M
BTN3A3
HLA-DQB1
HLA-F
HLA-C
TRIM5
IFI16
ZC3HAV1
TLR3
IFIT2
SP100
ISG20
TNFSF10
TNFSF10
PML
BIRC3
APOBEC3G
MYD88
ADAR
HLA-G
IL7
SP100
MIR5193///UBA7
OAS2
PSMB8
IFI16
TRIM21
STAT2
IFI30///PIK3R2
IFI16
APOL1
SP100
HLA-F
HLA-G
HLA-G
CASP1
HLA-E
HLA-E
MIR5193///UBA7
CASP1
HLA-E
APOBEC3F///APOBEC3G
RSAD2
OAS1
USP18
MX2
IFIT3
DDX58
IRF7
TRIM22
STAT1
OAS3
OAS2
IFIH1
HLA-B
CTSS
CFH
STAT1
IRF9
HERC5
OASL
CFH///CFHR1
HLA-B
HLA-C
C1S
STAT1
ISG20
SP100
EIF2AK2
IFIT5
GBP1
GBP1
SP100
HLA-F
EIF2AK2
DHX58
IFIT5
IFIH1
BST2
HLA-C
IFITM2
IFITM3
HLA-B
HLA-C
DDX60
AIM2
IFI35
STAT1
STAT1
SAMHD1
OASL
STAT1
UBE2L6
[24h, IFN]
[24h, untreated]

Supp. Figure 3 D

LNT-229
GS-2 innate immune response
GO:0045087

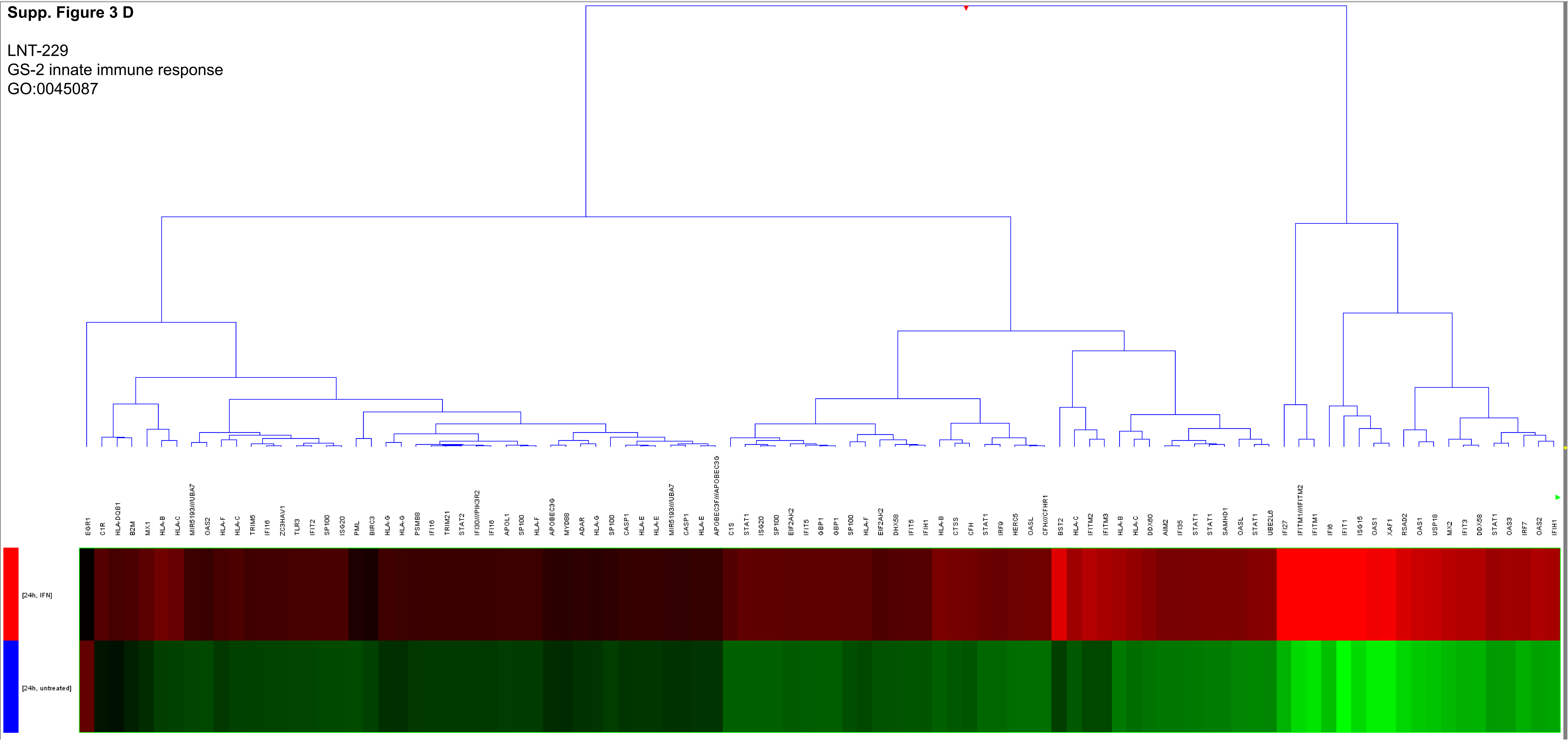

Supplement: S3 Fig — GS-2 (A, B) and LNT–229 (C, D) cells were treated with IFN-β (300 U/ml, 24 h) and the changes in the transcriptome were assessed using Affymetrix chip-based expression profiling. Results are presented as heat map. (PDF) [file pone.0139603.s003.pdf]
