# Supplementary material for: Interferon-β Modulates the Innate Immune Response against Glioblastoma Initiating Cells
Source: PLoS One. 2015 Oct 6;10(10):e0139603. doi: 10.1371/journal.pone.0139603 (PMC4595134; doi:10.1371/journal.pone.0139603)
Supplement: S1 Table — LNT–229, GS-2 or GS-9 cells were treated with IFN-β (300 U/ml, 24h) and the changes in the transcriptome were assessed using Affymetrix chip-based expression profiling. Immune regulatory genes were identified according to Biological Process Ontology Guidelines. Results are summarized in a table, showing the official gene symbol, full name, Probe set ID (= Gene accession number) and the grade of up-regulation compared to untreated controls. (PDF) [file pone.0139603.s004.pdf]

Supp. Table 1

		LNT-229		GS-2		GS-9	
symbol	full name	Probe Set ID	x-fold up	Probe Set ID	x-fold up	Probe Set ID	x-fold up
AIM2	absent in melanoma 2	206513_at	6.96	206513_at	4.16		
APOBEC3F	apolipoprotein B mRNA editing enzyme	214995_s_at	2.32	214995_s_at	4.40	214995_s_at	2.02
APOBEC3G	apolipoprotein B mRNA editing enzyme	204205_at	2.04	204205_at	6.25	204205_at	5.37
APOL1	apolipoprotein L, 1	209546_s_at	2.69	209546_s_at	2.45		
B2M	beta-2-microglobulin	216231_s_at	2.34			216231_s_at	2.21
BST2	bone marrow stromal cell antigen 2	201641_at	10.18	201641_at	6.98	201641_at	51.00
C1R	complement component 1, r subcomponent	212067_s_at	2.36	212067_s_at	5.18	212067_s_at	2.61
C1RL	complement component 1, r subcomponent-like			218983_at	3.03		
C1S	complement component 1, s subcomponent	208747_s_at	4.29	208747_s_at	5.24	208747_s_at	4.23
C3	complement component 3			217767_at	3.08	205476_at	13.61
C4A	complement component 4A			208451_s_at<br>214428_x_at	3.54<br>3.32		
CCL2	chemokine (C-C motif) ligand 2			216598_s_at	2.31		
CCL20	chemokine (C-C motif) ligand 20			205476_at	7.61		
CCL5	chemokine (C-C motif) ligand 5					204655_at	2.10
CCL8	chemokine (C-C motif) ligand 8			214038_at	11.18		
CCR1	chemokine (C-C motif) receptor 1			205099_s_at	2.93		
CFB	complement factor B			202357_s_at	9.08		
CFH	complement factor H	213800_at	5.12	213800_at	10.52		
CFHR1	complement factor H-related 1	215388_s_at	6.07	215388_s_at	8.11		
CTSS	cathepsin S	202902_s_at	5.41	202902_s_at<br>202901_x_at	9.19<br>4.65	202902_s_at	2.33
CXCL1	chemokine (C-X-C motif) ligand 1			204470_at	2.56		
CXCL10	chemokine (C-X-C motif) ligand 10			204533_at	222.89	204533_at	170.83
CXCL11	chemokine (C-X-C motif) ligand 11			211122_s_at<br>210163_at	189.96<br>90.86	211122_s_at<br>210163_at	22.29<br>13.78
CXCL2	chemokine (C-X-C motif) ligand 2			209774_x_at	7.03		
CXCL3	chemokine (C-X-C motif) ligand 3			207850_at	4.40		
CXCL5	chemokine (C-X-C motif) ligand 5			215101_s_at<br>214974_x_at	2.11<br>2.11		
CXCL6	chemokine (C-X-C motif) ligand 6			206336_at	3.43		

DDX58	DEAD (Asp-Glu-Ala-Asp) box polypeptide 58	218943_s_at	18.24	218943_s_at	43.15		
		219364_at	3.86	219364_at	5.55	218943_s_at	51.44
ENPP2	ectonucleotide pyrophosphatase/phosphodiesterase 2			210839_s_at	2.30		
ERAP1	endoplasmic reticulum aminopeptidase 1	210385_s_at	2.26	209788_s_at<br>210385_s_at	2.13<br>2.04	209788_s_at<br>210385_s_at<br>214012_at	3.34<br>2.85<br>2.60
EREG	epiregulin			205767_at	11.12		
GBP2	guanylate binding protein 2, interferon-inducible			202748_at	4.76		
HERC5	hect domain and RLD 5	219863_at	5.93	219863_at	7.41	219863_at	24.41
HLA-A	major histocompatibility complex, class I, A			215313_x_at<br>213932_x_at	2.42<br>2.24	215313_x_at<br>213932_x_at	3.24<br>2.86
HLA-A /-F /-J	major histocompatibility complex, class I, A / F / J			217436_x_at	3.03	217436_x_at	3.40
HLA-B	major histocompatibility complex, class I, B	211911_x_at<br>209140_x_at	5.86<br>9.95	211911_x_at<br>208729_x_at	4.97<br>4.62	209140_x_at<br>211911_x_at	18.25<br>6.93
		208729_x_at	4.15	209140_x_at	4.25	208729_x_at	5.71
HLA-C	major histocompatibility complex, class I, C	216526_x_at<br>214459_x_at<br>211799_x_at<br>208812_x_at	7.44<br>3.85<br>3.15<br>8.07	211799_x_at<br>214459_x_at<br>216526_x_at<br>208812_x_at	4.54<br>4.07<br>3.76<br>3.76	208812_x_at<br>216526_x_at<br>214459_x_at<br>211799_x_at	10.81<br>9.40<br>5.60<br>4.23
HLA-DQB1	major histocompatibility complex, class II, DQ beta 1	209480_at	2.04				
HLA-E	major histocompatibility complex, class I, E	217456_x_at<br>200905_x_at<br>200904_at	2.41<br>2.33<br>2.43	200904_at<br>200905_x_at<br>217456_x_at	9.99<br>7.42<br>4.49	200905_x_at<br>200904_at<br>217456_x_at	15.65<br>11.69<br>9.94
HLA-F	major histocompatibility complex, class I, F	221978_at<br>221875_x_at<br>204806_x_at	2.66<br>3.78<br>2.79	221978_at<br>221875_x_at<br>204806_x_at	5.95<br>4.45<br>4.10	221875_x_at<br>204806_x_at	4.39<br>3.71
HLA-G	major histocompatibility complex, class I, G	211530_x_at<br>211529_x_at<br>211528_x_at	2.12<br>2.35<br>2.50	211528_x_at<br>211529_x_at<br>210514_x_at<br>211530_x_at	3.69<br>3.62<br>3.37<br>2.89	211529_x_at<br>211528_x_at<br>210514_x_at<br>211530_x_at	4.12<br>4.10<br>4.03<br>3.13
ICAM1	intercellular adhesion molecule 1			202638_s_at<br>202637_s_at	3.78<br>2.98	202638_s_at	3.28
IDO1	indoleamine 2,3-dioxygenase 1			210029_at	11.24		
IFI35	interferon-induced protein 35	209417_s_at	6.91	209417_s_at	12.27	209417_s_at	27.73
IFI44L	interferon-induced protein 44-like	204439_at	246.84	204439_at	15.18	204439_at	293.04

IFI6	interferon, alpha-inducible protein 6	204415_at	45.96	204415_at	16.84	204415_at	77.03
IFIH1	Interferon induced with helicase C domain 1	219209_at<br>216020_at	15.03<br>3.94	219209_at<br>216020_at	35.00<br>5.57	219209_at<br>216020_at	102.61<br>6.96
IFITM1	interferon induced transmembrane protein 1 (9-27)	214022_s_at<br>201601_x_at	155.57<br>136.97	201601_x_at<br>214022_s_at	14.46<br>12.49	214022_s_at<br>201601_x_at	172.93<br>158.53
IFITM2	interferon induced transmembrane protein 2 (1-8D)	201315_x_at<br>212203_x_at	7.75<br>7.05	201315_x_at<br>212203_x_at	4.31<br>3.38	201315_x_at<br>212203_x_at	18.31<br>57.42
IFITM3	interferon induced transmembrane protein 3 (1-8U)					208436_s_at	16.87
IL12A	interleukin 12A			207160_at	2.64		
IL15	interleukin 15			205992_s_at	2.32		
IL1R1	interleukin 1 receptor, type I			202948_at	2.10		
IL6	interleukin 6 (interferon, beta 2)			205207_at	6.92		
IL7	interleukin 7	206693_at	2.58	206693_at	2.90		
IL8	interleukin 8			211506_s_at<br>202859_x_at	11.51<br>10.47		
IRF7	interferon regulatory factor 7	208436_s_at	14.92	208436_s_at	8.62		
LIF	leukemia inhibitory factor			205266_at	2.12	205266_at	2.73
LY75	lymphocyte antigen 75			205668_at	2.20		
MR1	major histocompatibility complex, class I-related			207565_s_at	2.45		
MYD88	myeloid differentiation primary response gene (88)	209124_at	2.01	209124_at	3.48		
NFIL3	nuclear factor, interleukin 3 regulated			203574_at	2.25		
NOD2	nucleotide-binding oligomerization domain containing 2			220066_at	4.19		
OAS1	2',5'-oligoadenylate synthetase 1, 40/46kDa	205552_s_at<br>202869_at	26.49<br>49.72	205552_s_at<br>202869_at	39.50<br>36.62	202869_at<br>205552_s_at	104.55<br>65.15
OAS2	2'-5'-oligoadenylate synthetase 2, 69/71kDa	206553_at<br>204972_at	2.84<br>15.39	204972_at<br>206553_at	39.86<br>8.21	204972_at<br>206553_at	23.02<br>6.06
OAS3	2'-5'-oligoadenylate synthetase 3, 100kDa	218400_at	13.11	218400_at	13.53	218400_at	10.53
OASL	2'-5'-oligoadenylate synthetase-like	210797_s_at<br>205660_at	6.03<br>8.16	205660_at<br>210797_s_at	40.60<br>28.92	205660_at<br>210797_s_at	13.03<br>12.43
PROCR	protein C receptor, endothelial			203650_at	2.06		
PSMB9	proteasome subunit, beta ype, 9					204279_at	21.91
PSMB10	proteasome subunit, beta type, 10			202659_at	2.60	202659_at	2.29
RGS1	regulator of G-protein signaling 1					216834_at	4.28
SAMHD1	SAM domain and HD domain 1	204502_at	7.64	204502_at	7.74	204502_at	9.53

SECTM1	secreted and transmembrane 1			213716_s_at	2.63		
SERPING1	serpin peptidase inhibitor, clade G (C1 inhibitor)			200986_at	3.14		
SIK1	salt-inducible kinase 1					208078_s_at	2.19
TAP1	transporter 1, ATP-binding cassette, sub-family B	202307_s_at	3.34			202307_s_at	10.23
TAP2	transporter 2, ATP-binding cassette, sub-family B			204769_s_at	2.77	204770_at<br>204769_s_at	2.27<br>2.26
TAPBP	TAP binding protein (tapasin)			208829_at	2.23	208829_at	2.70
TAPBPL	TAP binding protein-like					218747_s_at<br>218746_at	2.03<br>2.01
TLR3	toll-like receptor 3	206271_at	3.13	206271_at	11.24	206271_at	21.72
TNFRSF14	tumor necrosis factor receptor superfamily, member 14			209354_at	2.28		
TNFSF10	tumor necrosis factor (ligand) superfamily, member 10	202688_at<br>202687_s_at	3.02<br>3.55	202688_at<br>202687_s_at	100.21<br>85.57	202687_s_at	4.02
				214329_x_at	62.83		
TRAFD1	TRAF-type zinc finger domain containing 1			35254_at	2.36		
TRIL	TLR4 interactor with leucine-rich repeats			205150_s_at<br>205151_s_at	4.00<br>3.80		
TRIM22	tripartite motif-containing 22	213293_s_at	12.73	213293_s_at	32.01	213293_s_at	172.76
VEGFA	vascular endothelial growth factor A	211527_x_at<br>210513_s_at<br>212171_x_at	3.39<br>3.14<br>2.47				
VNN1	vanin 1	205844_at	5.19				
